# The Influence of the Modulation Index on Frequency-Modulated Steady-State Visual Evoked Potentials

**DOI:** 10.3389/fnhum.2022.859519

**Published:** 2022-03-10

**Authors:** Alexander M. Dreyer, Benjamin L. A. Heikkinen, Christoph S. Herrmann

**Affiliations:** ^1^Applied Neurocognitive Psychology Lab, Department of Psychology, European Medical School, Carl von Ossietzky University, Oldenburg, Germany; ^2^Experimental Psychology Lab, Department of Psychology, European Medical School, Cluster for Excellence “Hearing for All”, Carl von Ossietzky University, Oldenburg, Germany; ^3^Neuroimaging Unit, European Medical School, Carl von Ossietzky University, Oldenburg, Germany; ^4^Research Center Neurosensory Science, Carl von Ossietzky University, Oldenburg, Germany

**Keywords:** steady-state visual evoked potentials (SSVEP), brain-computer interface (BCI), electroencephalography (EEG), frequency modulation, user experience

## Abstract

Based on increased user experience during stimulation, frequency-modulated steady-state visual evoked potentials (FM-SSVEPs) have been suggested as an improved stimulation method for brain-computer interfaces. Adapting such a novel stimulation paradigm requires in-depth analyses of all different stimulation parameters and their influence on brain responses as well as the user experience during the stimulation. In the current manuscript, we assess the influence of different values for the modulation index, which determine the spectral distribution in the stimulation signal on FM-SSVEPs. We visually stimulated 14 participants at different target frequencies with four different values for the modulation index. Our results reveal that changing the modulation index in a way that elevates the stimulation power in the targeted sideband leads to increased FM-SSVEP responses. There is, however, a tradeoff with user experience as increased modulation indices also lead to increased perceived flicker intensity as well as decreased stimulation comfort in our participants. Our results can guide the choice of parameters in future FM-SSVEP implementations.

## Introduction

Rhythmic light stimulation with a constant frequency elicits steady-state visual evoked potentials (SSVEPS) measurable with electroencephalography (EEG) over the occipital cortex (Silberstein, 1995; Notbohm et al., 2016). Frequency-modulated (FM)-SSVEPs have been introduced as a novel stimulation method for brain-computer interfaces (Dreyer and Herrmann, 2015) which uses a sinusoidal carrier modulated by a second frequency as stimulation signals to evoked SSVEP responses at the difference between carrier and modulation frequency. Traditionally used SSVEPs can be evoked in a wide range of frequencies (Herrmann, 2001) and different stimulation parameters have been investigated in depth (Ng et al., 2012; Kuś et al., 2013; Norcia et al., 2015) along with detailed investigations into brain-computer interface (BCI) applications (Vialatte et al., 2010) as well as their potential user base (Allison et al., 2010). In contrast, detailed comparisons for different stimulation parameters that are required for FM-SSVEP stimulation are missing. In prior research, FM-SSVEPs have been shown in a variety of frequencies with simultaneously flickering light sources (Dreyer et al., 2017) as well as in different attention and perceptibility conditions (Lingelbach et al., 2021a). Other FM-SSVEP stimulation parameters have not been investigated in detail, especially the modulation index. Dreyer and Herrmann (2015) used a modulation index of 0.5 whereas more recent studies used a modulation index of 2.0 (Dreyer et al., 2017; Lingelbach et al., 2021b). The intuitive reasoning behind this increase was that it elevates the signal power in the targeted frequency in the stimulation signal and should therefore equally increase the measured FM-SSVEP power in EEG electrodes over occipital cortex. While this sounds straight-forward, an empirical investigation of this effect is so far missing.

Additionally, an argument against the use of SSVEPs in general is how rhythmic light stimulation affects the potential users. Constant attention to flickering light sources results in user discomfort (Ortner et al., 2011) and increased fatigue levels (Cao et al., 2014). Such effects can be reduced with high frequency stimulation paradigms (Sakurada et al., 2015) but high frequency stimulation can in turn reduce BCI performance (Volosyak et al., 2011). Other methods like amplitude modulated signals (Chang et al., 2014) or specific low frequency signals (Jiang et al., 2022) have been reported to increase user experience as well. In addition, we have previously shown that FM stimulation signals with a high carrier frequency are less perceptible (Dreyer and Herrmann, 2015) and more comfortable (Dreyer et al., 2017) than traditionally used stimulation signals. Similar to the FM-SSVEP power mentioned above, the effect of the modulation index on such subjective user experience factors has to the best of our knowledge not been empirically investigated.

In the current study, our goal was to investigate the effect of changing the modulation index on FM-SSVEP power to guide parameter decisions in future research. We tested four different modulation conditions in three different frequencies. Our results reveal that increasing the signal power in the target frequency through the modulation index does in fact lead to increased FM-SSVEP power in the EEG. In addition, we found a tradeoff between increased modulation index and user-comfort during the stimulation.

## Materials and Methods

### Participants

We recorded the EEG of 14 participants (11 female) with a mean age of 24.4 years, who were paid for participation. All participants had normal or corrected-to-normal vision. They reported not to have ever suffered from epilepsy and were informed about the risk of seizures in epileptics due to the visual stimulation. All participants gave their written informed consent and the overall study procedures was approved by the ethics committee of the University of Oldenburg.

### Stimuli

We used a green LED with a diameter of 1 cm to visually stimulate the participants and evoked the FM-SSVEPs. The LED was placed about 50 cm in front of the participants’ eyes, consequentially covering around 1.15° of the visual field. It was placed on top of a black plastic frame against a black cloth screen. In order to drive the LED, we used a custom signal amplifier connected to a digital-to-analog converter (NI USB-6229 BNC, National Instruments, Austin, Texas, United States) with a 10 kHz sampling rate which was fed by a stimulation computer running MATLAB (The MathWorks Inc., Natick, MA, United States). To generate the driving signal we used the same formular used in previous FM-SSVEP publications (Dreyer and Herrmann, 2015):


signal=A+F⁢V*sin⁢{2*π*Fc*t+[M*sin⁡(2*π*Fm*t)]}.

**FIGURE 1:**
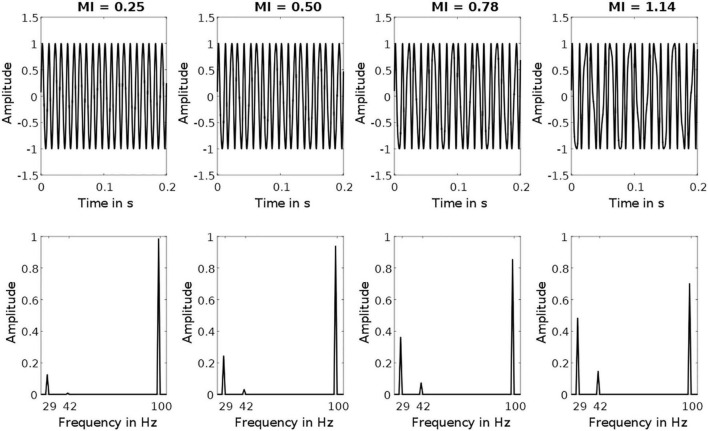
Exemplary Stimulation Signals. Top row depicts frequency-modulated signals with a carrier frequency of 100 Hz and a modulation frequency of 71 Hz. Bottom row depicts the corresponding amplitude spectra. Each column depicts a signal with one of the four different modulation indices used. Note that target frequency amplitudes at 29 Hz increase with increasing modulation indices (from left to right: 0.12, 0.24, 0.36, 0.48).

Here, A is the DC bias (2.5 V), FV is the flicker voltage span (1.8 V), Fc is the carrier frequency (100 Hz), Fm is the modulation frequency (71, 74, 77 Hz), M is the modulation index (0.25, 0.5, 0.78, 1.14), and t is the time vector. Parenthesized values represent the values used for stimulation. Note that the difference between Fc and Fm is where the FM-SSVEPs signals are to be expected (23, 26, 29 Hz). The signal amplitude in these bands depends on the given modulation index. Figure 1 depicts exemplary stimulation signals with their respective frequency spectra. The modulation index values we chose were based on the signal amplitude in the target frequency, with the amplitude in the higher conditions (0.5, 0.78, 1.14) being multiples of the amplitude in the lowest conditions (0.25). For the exemplary stimulation signal presented in Figure 1, the target amplitudes using the four modulation indices are 0.12, 0.24, 0.36, and 0.48 V.

All stimulation signals were tested and recorded with a photodiode to assure proper transfer of the signal generated in MALAB to the LED and that there was no clipping at the lower / upper boundaries of the brightness of the actual light output of the LED.

### Data Acquisition and Experimental Procedure

The recordings took place in an electrically shielded, dimly lit recording chamber. We used a BrainAmp EEG amplifier and the software Brain Vision Recorder (Brain Products GmbH, Gilching, Germany) for EEG acquisition. The sampling rate was 1000 Hz. Thirty-two electrodes (10-10 system locations: FP1, FP2, F7, F3, Fz, F4, F8, FT9, FC5, FC1, FC2, FC6, FT10, T7, C3, Cz, C4, T8, CP5, CP1, CP2, CP6, POz, P7, P3, Pz, P4, P8, O1, O2, and Oz) attached to size-appropriate EEG caps, including one EOG electrode below the right eye were recorded. Additionally, one reference electrode was placed on the tip of the nose and FCz was used as ground.

The participants were instructed to focus their gaze and attention on the center of the LED during the whole stimulation period. The twelve stimulation conditions (3 frequencies × 4 modulation indices) were presented in a randomized order. Each individual condition lasted 110 s after which the participants gave oral feedback using a 5-point Likert-Scale on the perceived flicker intensity (1 – no flicker to 5 – strong flicker) and their comfort during the stimulation (1 – very uncomfortable to 5 – very comfortable) which was noted by the experimenter.

### Data Analysis

**FIGURE 2:**
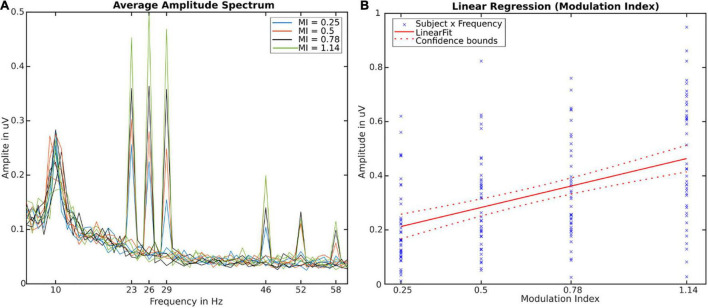
**(A)** Amplitude Spectra averaged over participants, channels and trials. Twelve individual lines depict stimulation conditions (frequency x modulation index). **(B)** Linear regression fit using modulation index (independent of frequency and subject) as predictor for the SSVEP-Amplitude [*R*^2^ = 0.2, *F*(1,166) = 42.55, *p* < 0.001]. Crosses depict all individual values for subjects × frequencies.

The EEG data was analyzed using MATLAB and the EEGLAB toolbox (Delorme and Makeig, 2004). The data was re-referenced to a common-average reference. All further analysis were done on four occipital channels of interest (O1, O2, Oz, and POz) in which FM-SSVEP responses can be expected. Data in these channels was zero-phase bandpass filtered between 1 Hz and 200 Hz. In order to avoid influence of the start or end of the stimulation, the first and last 5 s of the data were discarded. The resulting 100 s of stimulation data were split into 1 s long epochs which were corrected by their respective means. For the average amplitude spectra (see Figure 2A), we averaged the data over all participants, epochs and channels, respectively, for each stimulation condition, and then calculated the fast-Fourier transform of the resulting SSVEPs. Signal-to-noise ratios (SNRs) in the target frequencies were calculated by dividing the amplitudes in the target frequencies by the average amplitude of the two neighboring frequencies (Bach and Meigen, 1999). As our main effect of interest is the influence of the modulation index on the FM-SSVEPs, we then averaged the data over the three target frequencies for the statistical analyses.

## Results

We were able to reliably evoked FM-SSVEPS in all three target frequencies with all four modulation indices (see Figure 2A) as well as in their first harmonics. The respective amplitudes and SNRs increased with increasing modulation index. Individual subject data can be found in the Supplementary Materials Data Sheet 1. The SNRs in increasing modulation index order and averaged over frequencies were 4.25, 5.41, 7.08, and 10.6. For individual frequencies, the minimal SNR was 3.0 and the maximum SNR was 13.77. This means we were able to evoke statistically significant FM-SSVEPs at all frequency x modulation index combinations, as based on the SNR estimation approach we used (Meigen and Bach, 1999), SNR significance thresholds are 2.82 (*p* = 0.05) / 4.55 (*p* = 0.01). Analysis of variance with a single within-subject factor design with repeated measure revealed significant main effects of the modulation index levels on FM-SSVEP SNR [*F*(3,39) = 10.73, *p* < 0.0001] as well as amplitude [*F*(3,39) = 18.68, *p* < 0.0001]. Post-hoc pairwise *t*-tests with false-discovery rate (FDR) adjusted *p*-values revealed that amplitudes in all combinations significantly (*p* < 0.01) differed from each other except for the difference between 0.5 and 0.78. All statistical comparisons can be found in the Supplementary Material Tables 1, 2. Additionally, we used a simple linear regression to fit the relationship between our modulation index values and the SSVEP amplitudes combined over frequencies (see Figure 2B) and found a significant relationship [*R*^2^ = 0.204, *F*(1,166) = 42.44, *p* < 0.0001].

These findings empirically confirm the intuitive notion that increased power in the targeted frequency in a frequency-modulated stimulation signal results in increased FM-SSVEP responses.

### Flicker Intensity and User Comfort

Averaged user experience data can be found in Table 1. The perceived flicker intensity increased with increasing modulation indices while the perceived stimulation comfort decreased with increasing modulation indices.

**TABLE 1:** User-experience data averaged over participants and frequencies.

Modulation index	Perceived flicker intensity (SD)	Perceived stimulation comfort (SD)
0.25	1.36 (0.36)	4.40 (0.49)
0.50	2.48 (0.69)	3.71 (0.58)
0.78	3.19 (0.79)	3.36 (0.58)
1.14	3.52 (0.50)	3.00 (0.64)

We used single within-subject factor designs with repeated measures (14 subjects × 4 modulation indices) for flicker intensity and stimulation comfort, respectively. Analysis of variance revealed highly significant main effects of the modulation index levels on perceived flicker intensity [*F*(3,39) = 74.13, *p* < 0.0001] as well as on stimulation comfort [*F*(3,39) = 22.46, *p* < 0.0001]. Post-hoc pairwise *t*-tests with false-discovery rate (FDR) adjusted *p*-values revealed that all combinations significantly (*p* < 0.05) differed from each other except for the comfort rating difference between 0.5 and 0.78. Combined with the FM-SSVEP results above, we found a tradeoff where increased modulation indices increase the signal SNR but in turn decrease user experience.

## Discussion

Our goal in the current study was to assess the effect of different values for the modulation index on the FM-SSVEP response as well as the user experience during stimulation. We used four different values for the modulation index with increasing values leading to elevated sideband power in the target SSVEP frequencies. Our results reveal that these increases translate to stronger FM-SSVEP responses. These stronger responses go along with a rise in the perceived flicker intensity as well as a decrease in comfort during the stimulation.

We provide the missing empirical evidence for this intuitive notion that has already guided the choice of the modulation index in prior literature (Dreyer et al., 2017). Regarding the FM-SSVEP response, it seems plausible to suggest the use of a modulation index which leads to a relatively large amplitude in the first FM sideband especially for BCI applications where a high performance is wanted. It has to be noted, however, that sideband amplitudes in FM signals do not constantly increase with increasing modulation indices, but actually decrease above a certain modulation index threshold (≈ 2), as the power gets distributed to additional sidebands. Exact sideband amplitudes can be calculated using Bessel functions.

Overall, we found visible FM-SSVEP peaks with all modulation indices in all tested frequencies (23, 26, 29 Hz) with significant SNRs (Meigen and Bach, 1999) even though the FM-SSVEP amplitudes were rather small. Prior research has shown that FM-SSVEPs can be reliably classified even at relatively small amplitudes (Dreyer et al., 2017). It has to be taken into account that our stimulus LED only covered a small part of the visual field. For actual BCI implementations, larger stimuli of at least 2° visual angle have been suggested (Ng et al., 2012). Additionally, we also found peaks at the first harmonics of the target frequencies which could be useful for classification approaches (Müller-Putz et al., 2005).

Our results also show that increasing FM-SSVEP power *via* the modulation index comes with the tradeoff of increased flicker perceptibility and decreased comfort. Depending on the goal of the implementation and the target population, such factors have to be considered. It is quite intriguing to see that the least intrusive stimulation signal (modulation index = 0.25) already results in significant FM-SSVEPs. When user comfort is the highest priority, tweaking other stimulation parameters, stimulation size for example, might be preferable. Whether an increase in FM-SSVEP power *via* larger stimuli would lead to a similar tradeoff with user experience or not remains to be tested in future research, however.

The current manuscript complements a growing body of research on FM-SSVEPs for which we now have evidence that:

- They reduce flicker perceptibility compared to traditional sinusoidal or rectangular stimulation while retaining SNRs in the target frequency (Dreyer and Herrmann, 2015).
- They can be classified reliably at variable frequencies even with multiple, simultaneously flickering light sources (Dreyer et al., 2017).
- They are perceived as more comfortable than traditionally used sinusoidal stimuli (Dreyer et al., 2017).
- They can be evoked reliably even with an intensity below the individual perceptibility threshold and in covert attention conditions without direct fixation of the flickering light source (Lingelbach et al., 2021a; Lingelbach et al., 2021b).
- Their amplitude, their perceived flicker intensity, as well as the user comfort during the stimulation are affected by the modulation depths *via* the modulation index (current manuscript).

Together, these findings provide a foundation for future FM-SSVEP implementations into BCI applications as well as neurophysiological research approaches.

## Data Availability Statement

The raw data supporting the conclusions of this article will be made available by the authors, without undue reservation.

## Ethics Statement

The studies involving human participants were reviewed and approved by Research Ethics Committee, University of Oldenburg, Germany. The patients/participants provided their written informed consent to participate in this study.

## Author Contributions

AD: study design, manuscript preparation, and data analysis. BH: manuscript preparation, data acquisition, and analysis. CH: study design, manuscript preparation, and study supervision. All authors read and approved the final manuscript.

## Conflict of Interest

The authors declare that the research was conducted in the absence of any commercial or financial relationships that could be construed as a potential conflict of interest.

## Publisher’s Note

All claims expressed in this article are solely those of the authors and do not necessarily represent those of their affiliated organizations, or those of the publisher, the editors and the reviewers. Any product that may be evaluated in this article, or claim that may be made by its manufacturer, is not guaranteed or endorsed by the publisher.
